# The Chemokine CXCL14-like Immunoreactivity Co-exists with Somatostatin, but not NPY in the Rat Dorsal Horn and Has Intimate Association with GABAergic Neurons in the Lateral Spinal Nucleus

**DOI:** 10.1267/ahc.20-00004

**Published:** 2020-10-20

**Authors:** Toshiharu Yamamoto, Kenichi Sasaguri, Natsuki Mizumoto, Hirohumi Suzuki

**Affiliations:** 1 Brain Functions and Neuroscience Division, Department of Oral Science, Graduate School of Dentistry, Kanagawa Dental University, Inaoka-cho 82, Yokosuka, Kanagawa 238–8580, Japan; 2 Department of Dentistry, Oral and Maxillofacial Surgery, Jichi Medical University, School of Medicine, Yakushiji 3311-1, Shimotsuke, Tochigi 329-0498, Japan; 3 Department of Biology, University of Teacher Education Fukuoka, Akamabunkyou-machi 1–1, Munakata, Fukuoka 811–4192, Japan

**Keywords:** CXCL14, chemokine, dorsal horn, spinal cord, rat

## Abstract

Recent studies have proposed that the chemokine CXCL14 not only has a chemotactic activity, but also functions as a neuromodulator and/or neurotransmitter. In this study, we investigated the distribution of CXCL14 immunoreactive structures in the rat spinal cord and clarified the association of these structures with somatostatin, glutamic acid decarboxylase (GAD; a marker for GABAergic neurons), and neuropeptide Y (NPY). CXCL14 immunoreactive fibers and puncta were observed in lamina II, which modulates somatosensation including nociception, and the lateral spinal nucleus of the spinal dorsal horn at cervical, thoracic, and lumber spinal cord levels. These CXCL14 immunoreactive structures were also immuno-positive for somatostatin, but were immuno-negative for GAD and NPY. In the cervical lateral spinal nucleus, CXCL14 immunoreactive puncta, which were also immuno-positive for somatostatin, existed along the proximal dendrites of some of GABAergic neurons. Together, these results suggest that CXCL14 contributes to the modulation of somatosensation in concert with somatostatin. Neurons targeted by the CXCL14 fiber system include GABAergic neurons located in the lateral spinal nucleus suggesting that CXCL14 with somatostatin can influence the GABAergic neuron function.

## I Introduction

Chemokines are bioactive peptides (8–14 kDa) that mediate chemotactic activity for leukocytes and lymphocytes. Differences in amino acid residues in the N-terminal region separate chemokines into 4 subfamilies: C, CC, CXC, and CX3C [11]. The CXC-subfamily is composed of 17 members, CXCL1–CXCL17, characterized by two cysteines interposing a single amino acid residue in the N-terminal region [55]. Furthermore, these CXCLs are subdivided into two subclasses, ELR^+^ and ELR^−^ based on the presence or absence of the “Glu-Leu-Arg” sequence at the N-terminal region [20]. Generally, ELR^+^ chemokines are potent promoters of angiogenesis, whereas ELR^−^ chemokines are potent inhibitors of angiogenesis [42]. CXCL14 is a member of the ELR^−^ chemokines isolated independently as BRAK [22], BMAC [40], or MIP-2γ [8] from human tumor cells. Kurth *et al*. [28] has been demonstrated that CXCL14 is a highly selective chemoattractant for blood monocytes, as assessed using the transwell migration assay. Different from other chemokines, CXCL14-like substance is localized in a wide range of nervous tissues and endocrine cells suggesting that the substance acts as a neuromodulator and/or a hormone [44, 45, 53].

Significant modulation of primary afferent information including pain can occur in the dorsal horn of the spinal cord [1, 5, 21, 27, 36, 47]. In particular, the superficial dorsal horn (laminae I and II) is the main terminal field of nociceptive afferents [34, 47]. These layers contain densely packed neurons (interneurons) with axons that remain mainly in the spinal cord [48]. Although interneurons in these layers are diverse morphologically and biochemically, these neurons can be divided into two broad classes: inhibitory (GABAergic and/or glycinergic) and excitatory (glutamatergic) neurons [17, 18, 48]. Inhibitory neurons may contribute to the prevention of specific types of pain and suppression of itch [37, 39, 49, 52] and excitatory neurons may contribute to the pain states and provide a convergent pathway for nociception [30, 54].

The lateral spinal nucleus (LSN) is situated ventrolateral to the superficial spinal dorsal horn and is a characteristic nucleus of rat, mouse, and guinea-pig, but not rabbit, cat, or human [19]. LSN neurons project to various supraspinal areas such as the thalamus, hypothalamus, telencephalon, amygdala, periaqueductal gray (PAG), tractus solitarius nucleus, and parabrachial nucleus [4, 6, 7, 12–14]. In addition, LSN neurons project to the spinal lamina I, II, V, and VII as propriospinal projections [23]. LSN neurons receive descending projections from the raphe nuclei, brain stem reticular formation neuclei, dorsal column nuclei, and PAG [9, 29]. Furthermore, LSN neurons receive various peptidergic inputs from local spinal cord neurons [10]. Based on these characteristic inputs and outputs, the LSN may be involved in the transmission and modulation of afferent input, including nociception [24].

In this study, we clarify the nature of CXCL14 immunoreactive fibers in the rat dorsal horn and LSN, and investigate the association of CXCL14-immunoreactive fibers with GABAergic neurons in these areas.

## II Materials and Methods

Male Sprague-Dawley rats (n = 7) were used for this study. All animal procedures were performed according to the guidelines established by the Institutional Animal Care and Use Committee of Kanagawa Dental University (permitted no. 19-026). At first, animals were anesthetized with an isoflurane inhalation solution (Pfizer, New York, NY, USA) and then deeply anesthetized with sodium pentobarbital (Kyouritsu Seiyaku Corporation, Tokyo, Japan) by intraperitoneal injection (30 mg/kg). The animals were transcardially perfused with 0.85% NaCl, and subsequently with 4% formaldehyde and 0.2% picric acid in 0.1 M sodium phosphate buffer (PB; pH 6.9). The spinal cord was dissected with vertebra and separated into cervical, thoracic, and lumber spinal cord regions by the ramification of dorsal roots evaluated by stereoscopic microscopy. Vertebra were removed from each level of spinal cord, and each spinal cord region was placed in the same fixative for 1 day at 4°C and, then, immersed in 20% sucrose solution. Cervical enlargement (levels 6 and 7), thoracic (levels 2 and 3) and lumber enlargement (levels 3 and 4) were sampled as representative of each spinal cord region. Cervical, thoracic, and lumber spinal cords were cut transversely into 20 μm-thick sections using a sliding microtome (LS-113; Yamato Kohki Industrial Co. Ltd., Asaka, Japan) equipped with a frozen stage (an order made instrument by Shinano Seisakujyo Co. Ltd., Tokyo, Japan). The frozen stage was frozen with dry ice and then tissue blocks were also frozen with dry ice. The sections were cut by a blade (C35; Feather Safety Razor Co. Ltd., Osaka, Japan) set in a blade holder (no. 160; Feather Safety Razor). For further studies, cervical spinal cord from levels 2 to 3 (C2–C3) and levels 4 to 5 (C4–C5) were sagittally and horizontally cut into 20 μm-thick sections. The sections were stored in 0.1 M PB (pH 7.4) containing 0.9% saline (PBS).

Immunostaining was carried out according to our routine methods [45, 53]. Briefly, the sections were washed in PBS overnight, and incubated with rabbit anti-human CXCL14 antibody (no. 500-P237; PeproTech Inc., Rocky Hill, NJ, USA) diluted to 0.5 μg/ml in PBS containing 1% bovine serum albumin and 0.3% Triton X-100 (PBS-BSAT) for 24 hr at 4°C. The antibody had been purified by affinity chromatography employing an immobilized human CXCL14 matrix (manufacturer’s description). After washing in PBS, the sections were incubated in biotinylated goat anti-rabbit IgG (BA-1000; Vector Laboratories, Burlingame, CA, USA) diluted to 1:100 in PBS-BSAT for 1 hr at room temperature. The sections were washed again in PBS and incubated for 30 min at room temperature with avidin-biotin-horseradish peroxidase complex (PK-6100; Vector Laboratories) diluted to 1:200 in PBS-BSAT. After a final wash in PBS, the sections were reacted with 0.02% 3, 3'-diaminobenzidine tetrahydrochloride (DAB) and 0.005% hydrogen peroxide in 0.05 M Tris-HCl buffer solution (pH 7.4). The sections were counterstained with thionine and coverslipped using Malinol (Muto Pure Chemicals, Tokyo, Japan). Control staining to evaluate the antibody specificity of the anti-CXCL14 antibody was carried out by omitting the antibody during the first incubation, and by incubating antibody pre-absorbed with recombinant human CXCL14 (5 μg/ml, no. 300-50; PeproTech Inc.). Analysis of staining profiles were performed using a light microscope (Eclipse 50i; Nikon, Tokyo, Japan).

To clarify the nature of CXCL14-immunoreactive fibers in the spinal cord, we performed double immunostaining with somatostatin and neuropeptide Y (NPY) using identical sections. Furthermore, to investigate the association of CXCL14-immunoreactive fibers with inhibitory neurons in the spinal cord, we also carried out double staining with glutamic acid decarboxylase (GAD), the enzyme responsible for the conversion of glutamic acid to gamma-aminobutyric acid (GABA), using identical sections. Mouse monoclonal antibody against somatostatin (GTX71935, GeneTex Inco., Irvine, CA, USA; 1:200 dilution in PBS-BSAT), sheep anti-NPY serum (AB1583; Chemicon International Inc., Temecula, CA, USA; 1:1,000 dilution in PBS-BSAT) and mouse monoclonal antibody against GAD67 (one of molecular forms of GAD) (MAB5406; Chemicon International Inc.; 1:500 dilution in PBS-BSAT) were utilized for double immunofluorescence staining. In cases of double staining of CXCL14 with somatostatin and GAD, CXCL14 was visualized by Alexa Fluor 555-labeled goat anti-rabbit IgG (Abcam, Cambridge, UK), while somatostatin and GAD were visualized by fluorescein conjugated donkey anti-mouse IgG (Abgent, San Diego, CA, USA). In a case of double staining of CXCL14 with NPY, CXCL14 was visualized by Alexa Fluor 488-labeled donkey anti-rabbit IgG (Abcam) and NPY was visualized by Alexa Fluor 555-labeled donkey anti-sheep IgG (Abcam). Some sections were counterstained with 4',6-diamidino-2-phenylindole (DAPI) to visualize cell nuclei. Control staining for fluorescent immunostaining was performed as follows. Sections were incubated with only each secondary antibody, namely, Alexa Fluor 555-labeled goat anti-rabbit IgG, fluorescein conjugated donkey anti-mouse IgG, Alexa Fluor 488-labeled donkey anti-rabbit IgG, or Alexa Fluor 555-labeled donkey anti-sheep IgG without primary antibodies. Analysis of staining profiles were performed using a light microscope (Nikon) equipped with proper fluorescence filter blocks, XF05-2 (Omega Optical, Inc., Brattleboro, VT, USA) for DAPI, DM505 (Nikon) for Alexa Fluor 488 and fluorescein, and DM565 (Nikon) for Alexa Fluor 555.

## III Results

**Fig. 1.**
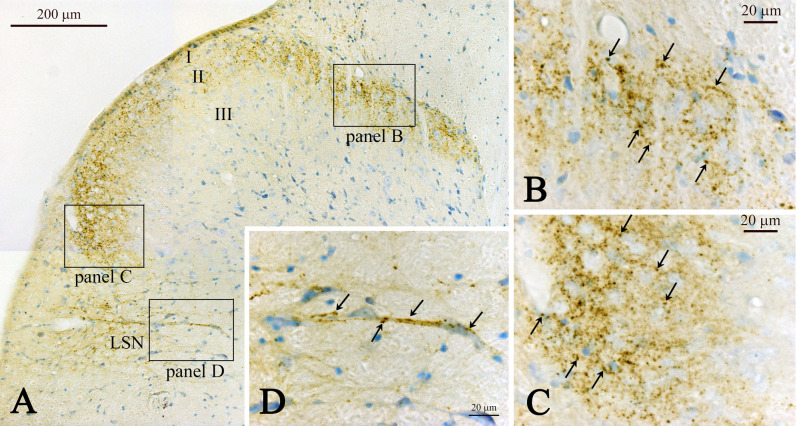
Representative low (**A**) and high (**B–D**) magnification images showing CXCL14 immunoreactive fibers in the cervical spinal cord. Boxed areas in **A** indicate magnified areas in **B–D**. (**D**) Note CXCL14 immunoreactive boutons are seen along with nerve processes of spindle-shaped neurons in the lateral spinal nucleus (LSN). Arrows in **B–D** indicate immunoreactive puncta. Abbreviations: I, lamina I; II, lamina II; III, lamina III. Bars = 200 μm (**A**) and = 20 μm (**B–D**).

**Fig. 2.**
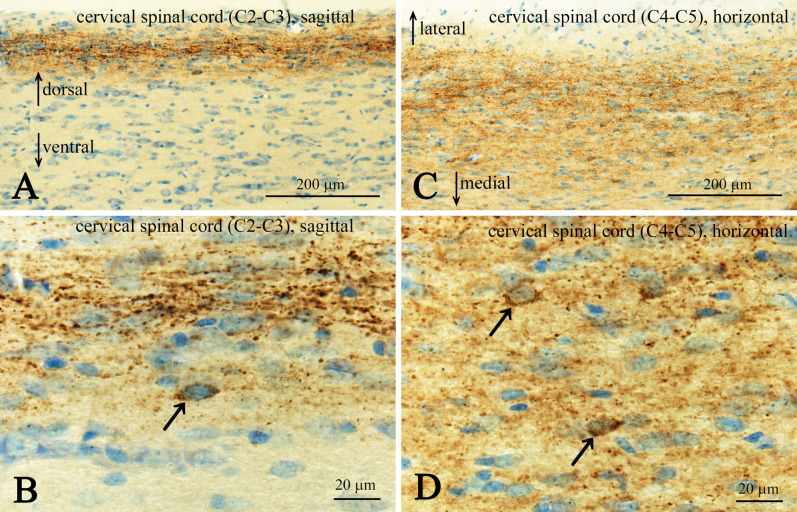
Representative low (**A** and **C**) and high (**B** and **D**) magnification images showing CXCL14 immunoreactive fibers in C2–C3 sagittal (**A** and **B**) and C4–C5 horizontal (**C** and **D**) sections of the cervical spinal cord. Arrows in **A** indicate dorsal and ventral directions and arrows in **C** indicate lateral and medial directions. Arrows in **B** and **D** indicate CXCL14 immunoreactive somata. Bars = 200 μm (**A** and **C**) and = 20 μm (**B** and **D**).

**Fig. 3.**
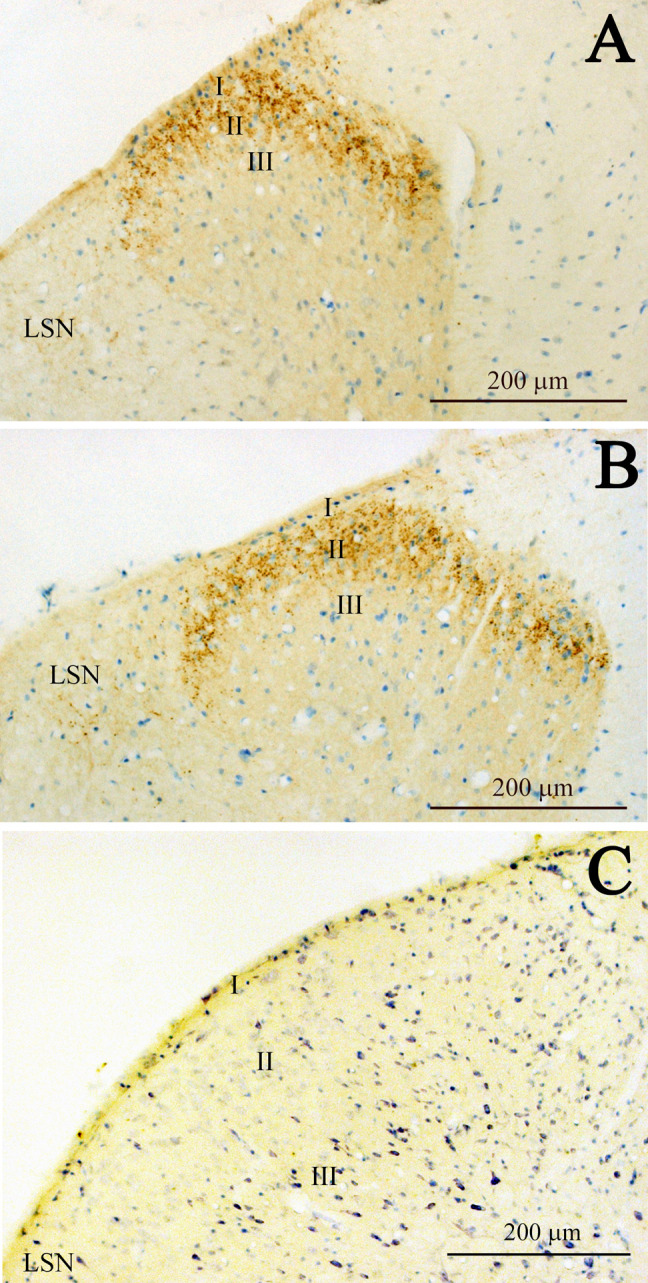
Representative low magnification images showing CXCL14 immunoreactive fibers in the thoracic (**A**) and lumbar (**B**) spinal cords. **C** shows effects of pre-absorption of the antibody with recombinant human CXCL14 on the staining profiles in the cervical spinal cord. Abbreviations: I, lamina I; II, lamina II; III, lamina III; LSN, lateral spinal nucleus. Bars = 200 μm (**A–C**).

In transverse sections, CXCL14-immunoreactive fibers were mostly restricted to lamina II and the lateral spinal nucleus (LSN) in the rat spinal cord (Fig. 1A). In the cervical spinal cord, CXCL14-immunoreactive puncta seemed to be localized around likely neuronal somata situated in lamina II (Fig. 1B, C). The density of immunoreactive puncta were similar in the medial (Fig. 1B) and lateral (Fig. 1C) subdivisions of lamina II, although the transition area of these two subdivisions showed a low density of immunoreactive puncta (Fig. 1A). In the LSN, CXCL14-immunoreactive fibers were sparser than that in lamina II and were observed as fibers in addition to puncta (Fig. 1). Characteristic profiles in this nucleus were that some of CXCL14-immunoreactive puncta, possibly terminal boutons, were localized along the proximal processes of neurons, of which processes were running in medio-lateral direction (Fig. 1A, D). In sagittal sections, CXCL14 immunoreactive structures in lamina II appeared as fibrous configurations (Fig. 2A, B). In addition, CXCL14 immunoreactive somata were sparsely seen in the inner layer of lamina II and their long axes were directed to the rostro-caudal direction (Fig. 2B). In horizontal sections, especially in the sections at the inner layer of lamina II, CXCL14 immunoreactive somata were scattered (Fig. 2C, D). CXCL14-immunoreactive configurations in the thoracic (Fig. 3A) and lumber (Fig. 3B) spinal cords were similar to those seen in the cervical spinal cord. However, terminal bouton-like structures were not observed in the LSN of the thoracic and lumber spinal cords (Fig. 3A, B). Pre-absorption of the CXCL14 antibody with recombinant CXCL14 abolished these staining profiles (Fig. 3C).

**Fig. 4.**
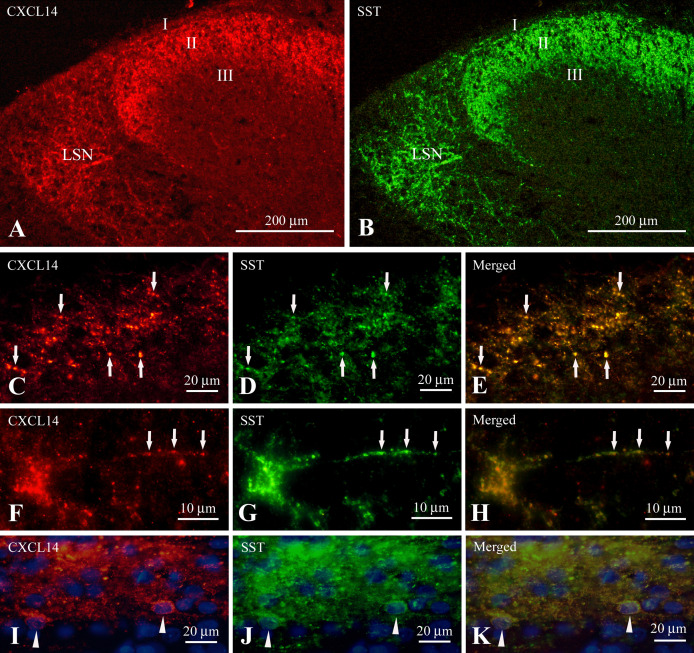
Representative low (**A** and **B**) and high (**C–K**) magnification images showing association of CXCL14-like immunoreactivity (**A, C, F** and **I**) with somatostatin (SST) immunoreactivity (**B, D, G** and **J**) in identical sections (**A** and **B**, **C** and **D**, **F** and **G**, **I** and **J**) of the cervical spinal cord. **I** and **J** are images from a sagittal section and counterstained with DAPI. Panels (**E**), (**H**), and (**K**) are merged images of (**C** and **D**), (**F** and **G**), and (**I** and **J**), respectively. Arrows in **C**, **D**, and **E** indicate that CXCL14 immunoreactive puncta are immuno-positive for SST in lamina II. Arrows in **F**, **G**, and **H** indicate that CXCL14-immunoreactive boutons along with immuno-negative nerve fibers in the lateral spinal nucleus (LSN) are also immuno-positive for SST. Arrowheads in **I**, **J**, and **K** indicate CXCL14 immunoreactive somata (in **I**) which are immuno-positive for SST (in **J**) in lamina II. Abbreviations in **A** and **B**: I, lamina I; II, lamina II; III, lamina III; LSN, lateral spinal nucleus. Bars = 200 μm (**A** and **B**), 20 μm (**C–E** and **I–J**), and 10 μm (**F–H**).

Somatostatin-immunoreactive fibers and puncta were mainly localized in lamina II and in the LSN (Fig. 4B). All CXCL14-immunoreactive puncta in lamina II were immuno-positive for somatostatin and all somatostatin immunoreactive puncta were immuno-positive for CXCL14 (Fig. 4C–E). In the LSN, all CXCL14 immunoreactive fibers and puncta were immuno-positive for somatostatin and all somatostatin immunoreactive fibers and puncta were immuno-positive for CXCL14 (Fig. 4A, B). A characteristic feature of the lateral spinal nucleus was that CXCL14 immunoreactive puncta, also immuno-positive for somatostatin, were localized along with proximal dendrites (Fig. 4F–H). Double staining of CXCL14 and somatostatin using horizontal sections indicated that CXCL14 immunoreactive somata were immuno-positive for somatostatin (Fig. 4I–K).

**Fig. 5.**
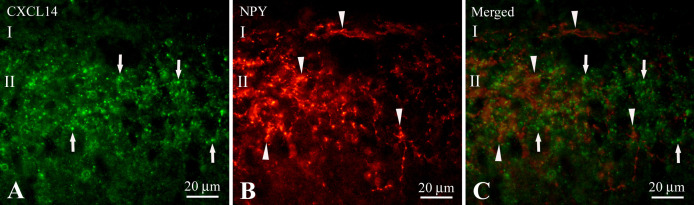
High magnification images showing association of CXCL14-like immunoreactivity (**A**) with NPY immunoreactivity (**B**) in an identical section of the cervical spinal cord. Panel (**C**) is a merged image of (**A**) and (**B**). Arrows in **A** and **C** indicate the CXCL14 immunoreactive puncta that are immuno-negative for NPY and arrowheads in **B** and **C** indicate the NPY immunoreactive fibers that are immuno-negative for CXCL14. Abbreviations: I, lamina I; II, lamina II. Bars = 20 μm.

Neuropeptide Y (NPY) immunoreactive fibers and puncta were seen in laminae I and II (Fig. 5B). NPY immunoreactive fibers and puncta were immuno-negative for CXCL14 (Fig. 5A–C).

**Fig. 6.**
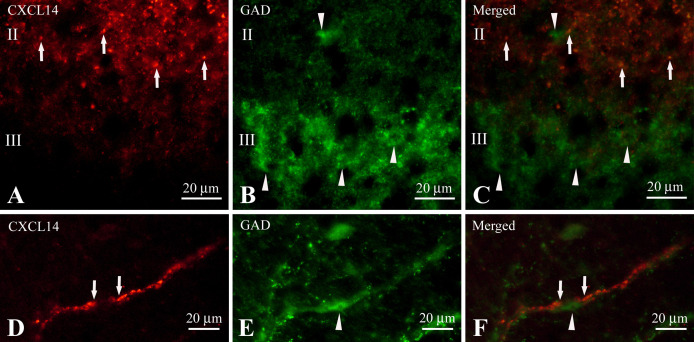
High magnification images showing associations of CXCL14 immunoreactive fibers and puncta (**A** and **D**) with GAD immunoreactive profiles (**B** and **E**) in identical sections (**A** and **B**, **D** and **E**). Panels (**C**) and (**F**) are merged images of (**A** and **B**) and (**D** and **E**), respectively. Arrows in **A** and **C** indicate CXCL14 immunoreactive puncta but immuno-negative for GAD in lamina II. Arrowheads in **B** and **C** indicate GAD immunoreactive soma but immuno-negative for CXCL14 in lamina II and III. Arrows in **D** and **F** indicate the CXCL14 immunoreactive puncta that are intimately associated with a GAD immunoreactive neuron (arrowheads in **E** and **F**) in the lateral spinal nucleus. Abbreviations in **A–C**: II, lamina II; III, lamina III. Bars = 20 μm.

Somata immunoreactive for glutamic acid decarboxylase (GAD), a marker for GABAergic neurons, were localized densely in lamina III and sparsely in lamina II (Fig. 6B). CXCL14-immunoreactive puncta in lamina II were immuno-negative for GAD and GAD-immunoreactive somata and fibers in laminae II and III were immuno-negative for CXCL14 (Fig. 6A–C). In the LSN, GAD-immunoreactive somata were also observed and some of these GABAergic neurons harbored CXCL14-immunoreactive puncta on their proximal dendrites (Fig. 6D–F). Counting 210 GABAergic neurons in the LSN of the cervical spinal cord, 13.3% of cells harbored CXCL14-immunoreactive puncta. However, we could not find such staining profiles in the LSN of the thoracic and lumber spinal cords. We could not find any immunoreactive fluorescence when sections were incubated with only fluorescence labeled secondary antibodies (not shown).

## IV Discussion

Chemokines are involved mainly in the activation and migration of leukocytes. However, novel evidence indicates non-immune functions of chemokines such as in neuron migration, neuromodulation, neuroendocrine regulation, and neurotransmitter-like actions [41, 43]. Previously our studies and others demonstrated that CXCL14 localizes in specific types of neurons and endocrine cells, namely pancreatic somatostatin-producing cells [2, 44], somatostatin-containing gastro-enteric endocrine cells, and the somatostatinergic nervous system in the alimentary tract [45], although, in the salivary glands, CXCL14 localizes in the NPYergic nervous system [46]. Furthermore, CXCL14 co-exists with hypothalamic oxytocin/vasopressin neurons [53] and dentate GABAergic neurons [3]. Physiologically, CXCL14 modulates the expression and maintenance of tyrosine hydroxylase (a marker enzyme of catecholaminergic neurons) in mesencephalic dopaminergic neurons [50], inhibits tonic and phasic effects of synaptically released GABA [3], and suppresses B-cell insulin secretion via the decrease of ATP levels [2]. All these results suggest the presence of other functions of CXCL14 instead of chemotactic function. Among co-existing substances, somatostatin, as reported here, is most frequent bioactive substance, suggesting an intimate relation with somatostatin, though detailed functions of CXCL14 in somatostatin system are not known. However, it is worthy to note that both somatostatin and CXCL14 have antitumor activities [25, 32, 33, 51]. Future studies may elucidate the association of these two peptides and their antitumor mechanism.

The dorsal horn of the spinal cord is the main site of termination for primary somatosensory afferent axons and contains the first synapses that transmit sensory information [1, 5, 47]. Within the dorsal horn, the substantia gelatinosa (lamina II), in particular, plays a key role in modulating somatosensation by selective inhibition of primary afferent inputs before they are transmitted to the brain [31]. Interneurons in laminae I–III are diverse in terms of their structure and function [54]. The interneurons can be divided into main groups: inhibitory and excitatory neurons [47]. Generally, somatostatinergic neurons are excitatory and NPYergic neurons are inhibitory [17, 54]. In the present study, we demonstrated that CXCL14 co-exists with somatostatin, but not NPY in lamina II, suggesting that CXCL14 involved in excitatory modulation of somatosensory inputs with somatostatin. Somatostatin fibers in the superficial dorsal horn mainly originate from propriospinal interneurons [35]. In fact, we demonstrated that intraspinal somatostatinergic somata are immuno-positive for CXCL14 suggesting that some of the CXCL14 immunoreactive fibers in lamina II originate from propriospinal interneurons. Alternatively, some of the CXCL14/somatostatin fibers may originate from the hypothalamus, where CXCL14 immunoreactive somata are present [53]. In fact, descending somatostatin fibers from the hypothalamus to the spinal cord have been documented [26]. The foregoing suggests that CXCL14 involved in the central modulation of primary afferent information including nociception in concert with somatostatin.

The lateral spinal nucleus (LSN) is a specific to rodents and not present in rabbit, cat, or human [19]. The electrophysiological properties of LSN neurons are similar to that of deep dorsal neurons rather than superficial dorsal horn neurons [24]. Neurons in the LSN project their fibers to various supraspinal areas, such as the thalamus, hypothalamus, amygdala, periaqueductal gray (PAG), tractus solitarius nucleus, and parabrachial nucleus [4, 6, 7, 12–14]. In addition, LSN neurons have propriospinal projections including those to spinal laminae I, II, V, and VII [23]. Although these neurons do not receive primary afferent terminals, they receive a variety of descending projections from the raphe nucleus, brain stem reticular formation nuclei, dorsal column nuclei, and PAG [9, 29], in addition to the propriospinal peptidergic terminals from the same or nearby segmental levels of the spinal cord [10, 16, 38]. In the present study, we demonstrated that CXCL14 co-exists with somatostatin in the fibers localized in the LSN suggesting that CXCL14 may contribute to somatostatin function related with the transmission and modulation of afferent information. Among LSN neurons, CXCL14-immunoreactive terminal boutons, which are also immuno-positive for somatostatin and intimately associate with some of GABAergic neurons, may influence GABAergic inhibitory function on the afferent inputs. The presence of GABAergic neurons in the LSN has been demonstrated [15].

In conclusion, we have demonstrated the co-existence of CXCL14 with somatostatin in the rat spinal cord suggesting that CXCL14 contributes in somatostatin function, namely, modulation of somatosensation including nociception.

## V Conflicts of Interest

The authors declare that there are no conflicts of interests regarding the publication of this paper.

## VI Acknowledgment

A part of this work was supported by JSPS KAKENHI grant number JP18K09842 to K. S.
